# The Perceptions of Telehealth Physiotherapy for People with Bronchiectasis during a Global Pandemic—A Qualitative Study

**DOI:** 10.3390/jcm11051315

**Published:** 2022-02-27

**Authors:** Annemarie L. Lee, Louise Tilley, Susy Baenziger, Ryan Hoy, Ian Glaspole

**Affiliations:** 1 Department of Allied Health Research, Cabrini Health, 154 Wattletree Road, Malvern, VIC 3144, Australia; ltilley@cabrini.com.au (L.T.); sbaenziger@cabrini.com.au (S.B.); 2 Department of Physiotherapy, School of Primary and Allied Health Care, Monash University, 45-47 Moorooduc Hwy, Frankston, VIC 3199, Australia; 3 Institute for Breathing and Sleep, Austin Health, 145 Studley Road, Heidelberg, VIC 3084, Australia; 4 Cabrini Health, 183 Wattletree Road, Malvern, VIC 3144, Australia; drryanhoy@gmail.com (R.H.); iglaspole@lasv.com.au (I.G.); 5 Allergy, Asthma and Clinical Immunology, Alfred Health, Commercial Road, Melbourne, VIC 3004, Australia

**Keywords:** bronchiectasis, telehealth, physiotherapy, COVID-19 pandemic

## Abstract

Physiotherapy is a core component of management for people with bronchiectasis and has predominantly been delivered in an in-person consultative format. With the global pandemic, a telehealth physiotherapy model of service evolved, but the perceptions and experiences from the consumer perspective of this service have not been evaluated. Participants who had a diagnosis of bronchiectasis and received a minimum of two telehealth physiotherapy sessions during the months of March 2020 to December 2020 at a private hospital were invited to take part in a semistructured interview. Interview transcripts were coded independently, with themes established by consensus from two researchers. In total, nine participants completed interviews (age range 44 to 83 years, 67% male), with four themes identified. Themes were initial mixed opinions and acceptance of telehealth physiotherapy as an alternate model, ease of use and limitations to the telehealth platform, enablers and barriers to physiotherapy service provision, and preferences for future models of telehealth physiotherapy beyond a pandemic. In the event of the continuation of telehealth physiotherapy services for people with bronchiectasis, the perceptions and experiences outlined by consumers could be applied to inform future modification of this model of service.

## 1. Introduction

Bronchiectasis is a chronic respiratory condition characterized by chronic cough with sputum production, fatigue, and recurrent respiratory infections [1]. To minimize symptoms and respiratory deterioration, clinical practice guidelines for bronchiectasis recommend the inclusion of personalized physiotherapy-led airway clearance and exercise therapy [2,3,4], with consistent reassessment [2,4]. Airway clearance therapies improve secretion clearance, and quality of life [5,6] and reduce the number of acute exacerbations [7], while exercise therapy improves exercise capacity, quality of life and leads to a longer time to an acute exacerbation [8]. Up until recently, the most common intervention model for providing the physiotherapy intervention to persons with bronchiectasis was through in-person consultations [9]. In the global COVID-19 pandemic, the delivery of telehealth has escalated to meet consumer requirements, with physiotherapy services rapidly transitioning to telehealth and frequently with limited preparation or staff training [10,11]. This included physiotherapy services for those with bronchiectasis [12]. Previous studies have highlighted the benefits of telehealth physiotherapy in overcoming challenges imposed by travel and transport [13,14,15]. Other advantages have been the increased flexibility with reduced disruption to work or daily routines [13,14]. During the pandemic, this model of care enabled physiotherapy to continue to be provided safely [14]. Preliminary reports in those with bronchiectasis revealed patient satisfaction for remote consultations [12]. However, perceptions and acceptance of this service from the patient perspective are unclear. As it is possible that the use of telehealth physiotherapy will continue in some form in the future [10,11], establishing this information is an important step which will direct and facilitate refinement of a telehealth physiotherapy model of care for this population. This study aimed to explore participant perceptions, acceptance, and experiences regarding telehealth physiotherapy provision in adults with bronchiectasis during a global pandemic.

## 2. Materials and Methods

### 2.1. Study Design

Prospective, qualitative study was conducted at Cabrini Health (Private Hospital). Ethics approval was granted by the Human Research Ethics Committee of Cabrini Health (02-05-11-20). Informed consent was obtained from all participants in the study.

### 2.2. Participants and Intervention

Individuals with bronchiectasis were considered eligible if they met the following criteria: Over the age of 18 years, diagnosis of bronchiectasis according to high-resolution computed tomography [2]; had received at least two sessions of outpatient telehealth physiotherapy in person at Cabrini Health in the past 12 months; and were able to provide written informed consent. Individuals were excluded if they had a primary respiratory diagnosis other than bronchiectasis or had an inability to communicate because of language skills, hearing, or cognitive impairment.

Telehealth physiotherapy sessions which were conducted by video conferencing technology (Zoom Video Communications Inc., San Jose, CA, USA, 2016) included prescription, modification, and supervision of airway clearance therapy and/or exercise therapies, including the use of video resources [16], discussion of strategies to manage adherence, and education and advice for self-management. Due to the rapid institution of social distancing recommendations and working from home directions in response to the global pandemic, the transition to telehealth physiotherapy service was prompt, to ensure delivery of ongoing physiotherapy. The physiotherapist did not receive specific training in telehealth physiotherapy but adapted her sessions according to recommendations [17], in consultation with colleagues and applying her clinical experience. Following the transition, as patients had already been referred and received outpatient physiotherapy at Cabrini Health, they were informed of the service adaption prior to their next scheduled appointment. All sessions were delivered on an individual basis. Each session was for a maximum duration of 60 min.

### 2.3. Data Collection

Participants were invited to take part in an individual semistructured interview. The interview explored participant experiences of telehealth physiotherapy, including the ease or challenges related to the experience. Interviews were conducted via telephone or videoconferencing (Zoom Video Communications Inc., San Jose, CA, USA, 2016). The interview schedule was informed by a previous qualitative investigation exploring telehealth physiotherapy in individuals with chronic respiratory conditions [18] as well as the existing body of literature [12,19]. Approximate duration of interviews was 30 min. Interviews were conducted by a researcher (A.L.L.) who was not involved in the delivery of the telehealth physiotherapy service and has received training in conducting semistructured interviews. Interviews were audio-recorded and transcribed verbatim and were continued until data saturation was reached [20].

Participants also completed a questionnaire related to satisfaction of the telehealth physiotherapy service, using the Telehealth Satisfaction Survey [21]. Demographics (age, gender, lung function) and bronchiectasis severity, using the Bronchiectasis Severity Index, a valid measure of bronchiectasis severity [22], were collated from the participants’ electronic medical record.

### 2.4. Data Analysis

Transcripts were stored and organized using a computer software program (QRS NVivo Version 12: QSR International, Doncaster, Australia). De-identified interview transcripts were examined by two researchers (ALL and LT), and line-by-line iterative thematic analysis [23] took place with descriptive codes devised to represent the data. Major themes were identified. The themes, including their description, were presented and discussed with a third reviewer (SB). Discussion of the themes took place between the three researchers until a consensus was reached. Quotations were extracted from the transcripts to provide supportive data for each theme.

## 3. Results

**Figure 1:**
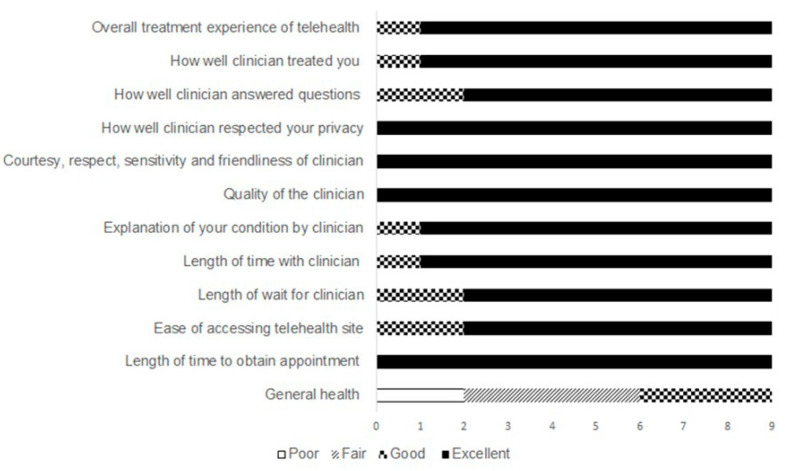
Participant satisfaction with telehealth sessions.

Out of a total of 13 individuals with bronchiectasis who received two sessions of telehealth physiotherapy over the period of April 2020 to December 2020, nine participants were recruited for this study. Prior to the adoption of the telehealth physiotherapy service, all participants had received between two and four sessions of in-person physiotherapy consultation. Following institution of the telehealth model, all participants had received maximum of two or three sessions of telehealth physiotherapy over the recruitment period. Demographic data of participants together with the airway clearance techniques and exercise activity undertaken at the time of the interview are outlined in Table 1. The disease severity ranged from 2 (mild) to 13 (severe), according to the Bronchiectasis Severity Index. Specifically, the individualized sessions included assessment of the participant’s current respiratory status, exercise tolerance, and adherence to a previously prescribed physiotherapy routine. Treatment sessions commonly incorporated modification of the performance of airway clearance techniques and introduction of a new or adjunct technique to facilitate secretion clearance as required. This was coupled with discussions outlining prescription and approaches to maximize adherence. Recommendations for exercise to improve exercise tolerance and education to manage common comorbidities within the scope of physiotherapy practice were also discussed [24]. General health ratings at the time of telehealth sessions were a mix of poor (22%), fair (44%), and good (33%). The level of overall satisfaction with the telehealth service was rated as excellent by 89% of participants, with similar or greater levels of satisfaction related to quality of treatment, clinician quality, ability of clinician to answer queries, courtesy, respect, and friendliness of clinician and for the consultation and quality of explanations (Figure 1). A total of four themes were identified.

### 3.1. Theme 1. Initial Opinion(s) about Telehealth Physiotherapy

Some participants highlighted their initial scepticism towards the concept of telehealth physiotherapy, due to a lack of confidence, concerns using unfamiliar technology, and questionable value of the session compared to in-person models.

“I just…to be honest, I was very hesitant at first because I didn’t know how it would work and what to expect…. The first time I started, that I was going to have it I was a little bit anxious….”

(P06)

“I wasn’t sure if I’d be able to handle it, do it, yeah technology side of things really, more than anything.”

(P06)

“I was willing to certainly give it a go and I just wondered how valuable it may be given that often there’s hand on treatment being given.”

(P07)

Others were very open to the idea, in view of the temporary cessation of in-person consultations secondary to lockdown restriction and strict social distancing requirements. An increased sense of safety from infection was reported.

“I think overall it was a good experience in relation to the other option which was not being able to see a physiotherapist at all.”

(P09)

“It’s much more efficient to do it that way and if there’s any residual concerns about the virus and contacts and things people might feel much safer doing it that way for a while.”

(P07)

“But for me, because of the bronchiectasis, I was hesitant about going into a hospital zone or wherever.”

(P05)

Others highlighted greater convenience, eliminating concerns linked to transport and geographical distance and greater time efficiency.

“I’d say that it’s a really good medium and it certainly worked well for me because, I can’t travel to Melbourne, so that’s important.”

(P03)

“I mean the best part about it for me, I didn’t have to travel to the centre for the treatment. I could do it from home, which certainly was very convenient. So that was good.”

(P07)

Participants highlighted that in general, the model was considered a satisfactory substitute, with no reduction in service quality, and the potential for fewer distractions.

“It’s an example where telehealth achieved exactly what would have been achieved in the room.”

(P09)

“You actually are looking at their faces quite close up on Zoom and I think there is even a stronger engagement with compared to sometimes when you’re sitting in a room with someone, you might be looking out the window because something distracts you….whereas on Zoom there’s almost a barrelling in of the focus.”

(P07)

### 3.2. Theme 2. Ease of Use and Limitations to the Telehealth Platform

The majority of participants reported the telehealth platform to be easy to use, with adequate clarity for audio and visual interactions.

“I’ve been using it a lot in the last 12 months, so I was pretty familiar with it.”

(P08)

“No, not challenging… I’d used it a bit socially and through University as well during the pandemic”

(P03)

“I think the basics of Zoom serve the purpose and I can’t think of features that would have enhanced the experience”

(P08)

Most participants reported that technical issues related to using the platform were minimal, noting the importance of a clinicians’ technological competence to troubleshoot issues to avoid frustration.

“It was easy. And there was one thing, when I first started, cause I hadn’t done a Zoom before and she just talked me through it, so it was quite, really simple.”

(P06)

“Obviously they would need to be confident in the software themselves, to be able to advise people having difficulties because sometimes these things do drop out and they can be a real impediment to relationship if it’s just frustrating.”

(P07)

Those participants who expressed frustration or dissatisfaction with the platform attributed this to equipment limitations and fatigue in virtual consultations.

“I have a really old computer. I think smart phones was better than the old computer on my desk…. It was especially hard hearing and the visuals”

(P01)

“I was using Zoom quite a bit, and I was getting sick of it. I carried that reluctance into the first session.”

(P01)

### 3.3. Theme 3. Enablers and Barriers to Physiotherapy Telehealth Service Provision

A mix of challenges with telehealth physiotherapy were reported, including the inability to incorporate a thorough assessment of a participants’ respiratory status. Some participants perceived this as a limitation, with dissatisfaction towards this component being curtailed, others considered it sufficiently substituted by other professionals.

“Well obviously you can’t listen to my chest or listen to my lungs, but I have a doctor for those…. There hasn’t really been any necessity to see someone else face-to-face”

(P06)

“It’s not going to be the same as a physical examination, but it was fine”

(P02)

“Without being in physical contact, she wasn’t able to do what I really get benefit from, which is, she checks my lungs with the stethoscope and that’s not something you can do remotely….”

(P09)

Similarly, participants reported that not all components of treatment were able to be undertaken. These limitations were apparent when hands-on correction or method of practice was desired but not provided.

“I really feel physio is usually a doing thing and it seemed to be difficult to do that part of it.”

(P04)

“It can’t do everything that a chest physio might like to do with a patient, something they like to bang and bash you a little bit. Like anything physical clearly that was not able to be achieved.”

(P07)

In contrast, other participants mentioned that selected functions within the platform were applied to facilitate assessment and treatment and optimize the interaction.

“Even being able to see the colour of phlegm over the camera because I happened to cough at that point and my physiotherapist said do you mind if I see the colour, so I was able to do that sort of thing”

(P09)

“… I got a good view of the demonstrations she did, so I could fully understand what she was trying to convey”

(P05)

Participants perceived that the success of a telehealth session was dependent on the healthcare professional’s skills. Participants’ confidence in their clinician, their expertise, and communication skills were paramount.

“You just have to feel comfortable and the person that’s giving it to you, you have to feel confident with them and they have to appear confident really….”

(P06)

“When I was doing it incorrectly, she corrected me on that and showed me what to do…. And it was no different to being with her actually, to be honest.”

(P05)

Some participants reported that the telehealth environment enabled them to keep their own records of the interaction, which may facilitate patient empowerment in their healthcare.

“I did make notes during the session, and I felt it was a very positive outcome and I think probably energized me to be a little more focused on my health.”

(P07)

“It allows both people to write things down. It’s not always so easy to have a pen and paper on your knee when you’re sitting in a room with the physio, but here you’ve got a desk in front of you, and you can make notes. I think that’s a plus as well.”

(P07)

### 3.4. Theme 4. Preferences for Future Models of Telehealth Physiotherapy beyond a Pandemic

With regards to the longer term, there were a mix of preference for future models of physiotherapy. Some participants preferred a hybrid model of physiotherapy, with the retention of an initial in-person consultation followed by telehealth.

“I think my experience wouldn’t have been as good if I hadn’t met her face-to-face beforehand, so we work out the baseline stuff.”

(P09)

“I think it would be a little bit difficult if it was just a telehealth service, I think, I’d certainly recommend initially a few, maybe one or two appointments in person.”

(P09)

A small portion of participants definitely preferred online only, expressing satisfaction with the service, with the benefits of less travel perspective and saving time.

“I’d be really quite happy to continue with the Zoom meetings because I think they’re very effective.”

(P03)

“I think they should use it as much as possible. It saves patients time and trouble, which they should try and do if they can.”

(P02)

“For many people, I suspect, it may be preferred because they don’t have to travel. If you have a half hour session and it takes half an hour, so it is actually much more efficient, then I think many people would prefer that and they may into the future.”

(P07)

In contrast, other participants affirmed an ongoing preference for in-person, to enable a complete grasp of all concepts discussed and a sufficiently thorough interaction.

“I’m probably a bit biased in a way, I sort of feel that physio is such, what would I say, physio is a doing type of thing, to a large extent, I don’t think it’s suitable for telehealth. If you want to have good sessions, I think it has to be face to face.”

(P04)

“Personally, I like the face to face much better. I have felt more satisfied when I’ve been on a face to face rather than on a Zoom link.”

(P04)

“I am really old school and I really like the face to face and touchy feely, sort of thing.”

(P01)

## 4. Discussion

This study outlines the consumers’ perspective of a telehealth physiotherapy service provided in an Australian private hospital during the COVID-19 pandemic. It is evident that there were a mix of initial perceptions of this service, but it was generally accepted as an alternative model in light of the imposed restrictions. Some participants found the platform employed easy to use; others experienced some challenges. A range of enablers and barriers to the telehealth physiotherapy service were identified and the preference for a future model appears to be mixed, including the hybrid combination of virtual and in-person consultations.

Scepticism towards telehealth services have been previously reported, with consumers cancelling, postponing consultations, or not adhering to the intervention when clinical interactions were pivoted to a telehealth format during the COVID-19 pandemic [25,26,27]. Professional acceptance of telehealth by patients and health care professionals has been noted to be challenging [28]. From the study findings, this may be influenced by the degree of confidence and familiarity with using the relevant technology from the consumers’ perspective [29]. The platform applied in the current study was associated with some difficulties for some participants, which mirrors earlier reports in older adults, including those with bronchiectasis, who were unfamiliar with this technology [18,26,30] This has been referred to as “computer anxiety” [31]. It highlights that it may be necessary to improve the awareness of telehealth services for consumers in order to foster positive attitudes and alleviate hesitation [32]. In addition, time allocated for training to ensure consumer familiarity with the technology may be a worthwhile investment [33]. It is noteworthy that some participants expressed minimal concern and a growth in confidence with sessions. Although this is not explored in the current study, an area of future research may focus on identifying factors which determine engagement with telehealth services in those with bronchiectasis.

A range of factors appeared to influence the acceptance of telehealth physiotherapy as an alternative option in selected participants. That it was considered to be a necessary adaption to ensure a continued service of delivery during the COVID-19 pandemic, when stay-at-home orders were stipulated, has been similarly reported in other populations [25,27,30]. The reduced burden of travel with telehealth services compared to in-person consultation has also been observed for those with chronic conditions [25,34], with lower reports of physical exhaustion in those with chronic respiratory conditions undertaking telehealth [30,35,36]. Reducing the risk of viral transmission during a pandemic [37] or the risk of infection linked to attending a healthcare facility [30], a particular concern for those with bronchiectasis, was also a perceived benefit of engaging with telehealth. These factors may influence the growth of telehealth physiotherapy in the future, both for consumers living locally and those at a geographical distance.

For some participants, views that the service provided was a replica of the in-person consultations has also been recently observed in studies of people with chronic conditions [26,38]. In those with cystic fibrosis, a telehealth physiotherapy model evaluated quality of life and physical activities levels and was considered a means to provide instruction and modification for airway clearance therapy with real-time feedback [35], with high levels of satisfaction with some services [25]. Greater focus and less socialization have been noted as benefits of virtual visits, which may enhance engagement for some participants [39]. Patients rate communication processes together with technology-based skills as the most important for telemedicine [33,40,41]. From a practical perspective, there is merit in equipment quality and placement when demonstrating or modifying the performance of treatment to support a telepresence [29]. The telehealth satisfaction survey results generally indicated a high degree of satisfaction with the service provided in the current study. This highlights that some of these strategies and methods which are included in the expert consensus of telehealth skills for health care professionals [33] were successfully adopted in the current study.

One of the noted constraints with telehealth physiotherapy was the elimination of physical contact between the therapist and patient [42]. As touch is considered one of the key distinguishing features which defines physiotherapy practice [43], a challenge of telehealth is the ability to create an alternative avenue for feedback and modification [44]. While medical personnel have reported minimal additional benefit related to auscultation for patients with respiratory disease [35], the absence of this tool during physiotherapy sessions was a concern for some participants in the current study. This, coupled with the elimination or substitution of assessment components which require physical examination, was a noted shortcoming, a perception previously observed in other populations [45,46]. This highlights the value in establishing expectations of a telehealth physiotherapy service delivery for consumers prior to an initial consultation.

The mixed views on future use of a telehealth physiotherapy model reflect reports in other populations, with some patients stating a preference for in-person consultations, while others anticipate use of telehealth beyond the pandemic [25,26,38]. It has been acknowledged that building rapport is facilitated when an existing real-life, in-person relationship is in place [35], a stance reflected by many participants in the current study. In addition, due to social distancing measures, patients have reported the need for inter-personal relationships and resuming human interaction with clinicians as part of their healthcare approach [25]. Telehealth provides the opportunity to complement in-person care and expand access to services via a hybrid model. What may be important in that hybrid model is retention of the initial in-person consultation to facilitate the establishment of a trusting relationship between the clinician and consumer [30,35]. With the possibility of the service model evaluated in the current study continuing to be offered in the future and considering the mixed perceptions from consumers, it may be important to initially determine which model is best suited to each individual. Where feasibility from the healthcare facility and resources for telehealth physiotherapy is apparent, this model could be offered as a hybrid or fully digital model for some consumers.

There are limitations in this study. With only a small number of participants diagnosed with bronchiectasis accessing the telehealth physiotherapy service during the study period, most participants had mild to moderate disease severity according to the Bronchiectasis Severity Index. The findings presented may not fully reflect the overall disease severity of those with bronchiectasis. All participants in this study had previously received in-person physiotherapy; the extent to which these perceptions apply for those whose initial consultation was via telehealth are yet to be determined. While not collated in the current study, the social status, level of education, and health literacy of participants has previously influenced engagement and acceptance of telehealth services [25,47,48]. The impact of these factors in those with bronchiectasis requires further exploration. While a mix of airway clearance techniques and approaches to exercise were prescribed for participants, there was a dominance of using devices rather than dependent techniques, and this may impact on the perceptions of the physiotherapy service.

## 5. Conclusions

In conclusion, people with bronchiectasis expressed initial scepticism and uncertainty towards a telehealth physiotherapy service, but there was general acceptance of this model, particularly with respect to complying with public health stay-at-home orders to minimize infection and continuing to receive treatment. A range of enablers and barriers to the physiotherapy service were identified, with a mix of preference for future telehealth models for physiotherapy bronchiectasis management. With the reports of an ongoing preference for in-person consultations from some participants, telehealth could be considered as an alternative to face-to-face sessions, but not imposed as a complete replacement for physiotherapy service.

## Figures and Tables

**Table 1:** Participant demographics.

Participant ID	Age (Years)	Sex	BSI	Airway Clearance Techniques	Exercise Routine
01	66	M	3	Aerobika^®^	Boxing, Swimming
02	68	M	5	NS inhalation, ACBT	Walking
03	68	F	3	Aeroclipse^®^, Aerobika^®^, HS/NS nebulization	Walking
04	83	F	13	Aerobika^®^, 6% HS nebulization	Walking
05	67	M	2	Aerobika^®^, NS nebulization, ACBT	Bike riding. Walking
06	82	F	7	ACBT, Flutter^®^, NS nebulization	Walking, Power fit machine
07	72	M	5	Flutter, IMT	Running, Gym
08	66	M	3	Aerobika^®^, ACBT	Bike riding. Walking
09	44	M	2	ACBT in positioning	Strength work. Pilates

ACBT—active cycle of breathing technique; BSI—bronchiectasis severity index; HS—hypertonic saline; IMT—inspiratory muscle training; NS—normal saline.

## Data Availability

Data are available for this study upon reasonable request.
